# Synergistic Interaction Between *Kazachstania humilis* and *Fructilactobacillus sanfranciscensis* Modulates Metabolic Reprogramming to Enhance Mantou Functionality in Liquid Sourdough

**DOI:** 10.3390/foods15101745

**Published:** 2026-05-15

**Authors:** Jingjing Liang, Beiqi Wu, Rong Guo, Jiaming Guo, Juanxia Wang, Guohua Zhang

**Affiliations:** 1 School of Life Science, Shanxi University, Taiyuan 030006, China; ljj322@sxu.edu.cn (J.L.); 18250808116@163.com (J.G.); 18636548656@163.com (J.W.); 2 School of Food Science and Technology, Jiangnan University, Wuxi 214122, China; bqwu@aliyun.com; 3 The Municipal Center of Comprehensive Inspection and Detection at Changzhi, Changzhi 046000, China; 15034270595@163.com

**Keywords:** liquid sourdough, *Kazachstania humilis*, *Fructilactobacillus sanfranciscensis*, starch digestibility, antioxidant properties

## Abstract

In this study, an acid-tolerant and high-fermentation performance strain of *Kazachstania humilis* (*K. humilis* 3-8) was screened from sourdough isolates and co-cultured with *Fructilactobacillus sanfranciscensis* (*F. sanfranciscensis* 5) to prepare liquid sourdough, which was further applied in mantou production. The effects on physicochemical properties, nutritional characteristics, and microbial interactions were investigated. *K. humilis* 3-8 exhibited strong gas production and acid tolerance, achieving a dough volume increase of 72.19% after 3 h fermentation. In co-culture, *F. sanfranciscensis* 5 maintained stable growth, while its metabolites significantly inhibited the growth of *K. humilis* 3-8 during mid-fermentation. The co-fermented dough showed decreased pH and increased total titratable acidity. Metabolomic analysis indicated enhanced carbohydrate metabolism and amino acid metabolism, suggesting carbon–nitrogen metabolic interactions between the two strains. When applied to Mantou production, the optimized co-culture system substantially enhanced product functionality, increasing resistant starch content by 76.7% (from 23.02% to 40.68%). Total phenolic content and antioxidant capacity were markedly enhanced. These findings elucidate complex microbial interactions governing sourdough ecosystems and establish a scientific foundation for the targeted improvement of traditional fermented cereal products through rational strain selection and process optimization.

## 1. Introduction

Sourdough constitutes a complex ecosystem arising from the spontaneous fermentation of grain flour and water, wherein metabolic interactions between lactic acid bacteria (LAB) and yeast not only govern fermentation kinetics but also confer superior textural and nutritional attributes upon the end products [1]. Traditional sourdough (Type I) is constrained by lengthy fermentation and operational complexity. Type II (liquid) sourdough resolves these issues via high dough yields and fermentation temperatures exceeding 30 °C, ensuring rapid, controllable industrial fermentation [2]. The sourdough ecosystem is dominated by LAB and yeasts, collectively comprising over 100 LAB species (predominantly *Fructilactobacillus sanfranciscensis*, *Lactiplantibacillus plantarum*, and *Levilactobacillus brevis*) and more than 80 yeast species (mainly *Saccharomyces*, *Kazachstania*, *Candida*, and *Torulopsis*) [3,4]. Yeast composition exhibits distinct regional patterns: French sourdoughs were primarily composed of *Kazachstania bulderi* and *K. humilis*, whereas German variants were primarily composed of *Pichia kudriavzevii* and *S. cerevisiae* [5,6]. Notably, *K. humilis* was identified as a prevalent yeast in traditional sourdough, typically coexisting with LAB and significantly influencing sourdough development and final bread characteristics [7].

Recent investigations demonstrate that synergistic fermentation of LAB and yeast significantly enhances the functional attributes of cereal-based products, encompassing textural, nutritional, and aromatic profiles [8,9,10]. Specifically, co-cultures improve bread and steamed bread quality by increasing specific volume and pore uniformity (*S. cerevisiae* to *Lpb. plantarum* ratio of 2:1) [11], while Tang et al. (2024) used *Saccharomycopsis fibuligera* combined with *Limosilactobacillus fermentum*, which facilitated wheat bran acidification and elevated volatile ethyl esters in Mantou, improving volume and texture [12]. Beyond structural modifications, these consortia augment nutritional functionality through specific strain combinations. Khan et al. [13] demonstrated that synergistic fermentation with *Lpb. plantarum*, *Lmb. fermentum*, and *S. cerevisiae* significantly elevated phenolic content, antioxidant capacity, and bioavailability in brown rice, while Zhao et al. revealed that co-cultures of active dry yeast with *Lactobacillus bulgaricus* and *Streptococcus thermophilus* enhanced water-soluble arabinogalactan, dietary fiber fractions, and water-holding capacity in wheat bran [14]. Mantou is a traditional Chinese steamed bread made from wheat flour dough fermented with yeast or sourdough and cooked by steaming rather than baking. It is widely consumed as a staple food in northern China.

Despite its established prevalence in traditional sourdough ecosystems and significant influence on fermentation kinetics and end-product characteristics, *K. humilis* has received limited systematic investigation compared to the extensively characterized *S. cerevisiae*, creating a critical gap in understanding the metabolic potential of non-conventional sourdough yeasts. To address this deficiency, this study screened *K. humilis* strains based on specific fermentation characteristics and established a synergistic co-culture system with *F. sanfranciscensis* for liquid sourdough production. By elucidating the metabolic interactions governing this consortium and quantifying their effects on Mantou quality and nutritional functionality, this work aims to expand the biotechnological repertoire of cereal fermentation beyond dominant model organisms and establish a scientific framework for the targeted industrialization of traditional fermented flour products.

## 2. Materials and Methods

### 2.1. Materials

A total of 28 *K. humilis* strains were obtained from Chinese sourdough and provided by the College of Life Science, Shanxi University, Taiyuan, China. These strains were stored at −80 °C for further use. Commercial *S. cerevisiae* was obtained from Lesaffre, Marcq-en-Barœul, France, strain number SY. Modified de Man, Rogosa and Sharpe (mMRS) medium and yeast extract peptone dextrose (YPD) medium were purchased from Beijing Aoboxing Biotechnology Co., Ltd. (Beijing, China). Wheat flour (Wudeli Fuqiang flour) was obtained from Wudeli Flour Group Co., Ltd. (Handan, China). 3,5-dinitrosalicylic acid (DNS), lactic acid, and acetic acid were purchased from Shanghai Macklin Biochemical Co., Ltd. (Shanghai, China).

### 2.2. Determination of Gas Production and Biomass Accumulation

The gas production characteristics of the different strains were determined by the Durham tube method Mou et al. [15]. The activated strains were inoculated into the YPD liquid medium with an inverted Durham tube at an inoculation volume of 2%, and the SY was set as the positive control. All tubes were incubated at 30 °C for 72 h. And gas accumulation in Durham tubes was recorded at 12 h intervals. The activated *K. humilis* was inoculated into the YPD medium at an inoculation volume of 2%, and incubated at 30 °C for 24 h. The optical density of each culture medium was measured to determine the growth density of different *K. humilis* strains within 24 h [16].

### 2.3. Physiological Functional Characteristics of Strains

#### 2.3.1. Determination of Fermentation Characterization

Following the method of Mou et al. [15] with minor modifications: Flour and sterile water containing *K. humilis* inoculum were mixed in graduated test tubes at a flour-to-water mass ratio of 4:5. After thorough mixing, tubes were incubated at 30 °C. Dough volume was recorded hourly, and dough volume growth rate (%) was calculated according to the following formula.

(1) $$\mathrm{Dough}\;\mathrm{volume}\;\mathrm{growth}\;\mathrm{rate}\;\left(\%\right)=\frac{H_{0}}{H}\times 100$$

where H_0_ is the height of the dough for the corresponding fermentation time, H is the initial height of the dough.

#### 2.3.2. Determination of Lactic and Acetic Acid Tolerance

Refer to the method of Xia et al., different concentrations of lactic acid (0.4–2%) and acetic acid (0.1–0.5%) were added to the YPD liquid medium and dispensed in centrifuge tubes, then the activated *K. humilis* was accessed at an inoculation volume of 2% [16]. The optical density was measured to characterize the growth density of *K. humilis* at different acid concentrations after 24 h incubation at 30 °C. The inhibition rate was calculated according to the following formula to characterize the tolerance of different strains to different concentrations of lactic acid and acetic acid.

(2) $$\mathrm{Inhibition}\;\mathrm{rate}\;\left(\%\right)=1-\frac{{OD}_{0}}{OD}\times 100$$

where OD_0_ is the optical density of bacterial solution in culture media with different concentrations of acid added, OD is the optical density of bacterial solution in YPD medium.

#### 2.3.3. Determination of pH and Growth Curve

YPD liquid medium was inoculated with activated cultures at a concentration of 1% and cultured at 30 °C for 48 h. Samples were taken at regular intervals. The pH and the optical density at 600 nm were measured at each sampling point using a pH meter (PHS-3C, INASE Scientific Instrument Co., Ltd., Shanghai, China) and a UV spectrophotometer (UV2600A, UNICO Instrument Co., Ltd., Shanghai, China).

### 2.4. Preparation of Liquid Sourdough

*F. sanfranciscensis* 5 and *K. humilis* 3-8 were statically cultured in MRS and YPD medium, respectively, at 30 °C for 36 h. Cultures were centrifuged (4500× *g*, 10 min, 4 °C) to collect cell pellets, which were washed twice with sterile saline and resuspended in distilled water. Liquid sourdough was prepared according to Table 1 formulations: Ingredients were aseptically mixed until homogeneous, sealed with parafilm, and fermented (30 °C, 80% RH).

### 2.5. Determination of Colony Counts of K. humilis and F. sanfranciscensis in Sourdough

Sourdough samples (10 g) were aseptically weighed and homogenized with 90 mL of sterile saline solution for 90 s to obtain a 1:10 (*w*/*v*) homogenate. Subsequently, 1 mL of the homogenate was subjected to a ten-fold serial dilution in sterile maximum recovery diluent to achieve appropriate concentrations. Aliquots (0.1 mL) from selected dilutions were spread-plated onto MRS medium supplemented with 100 mg/L actinomycin and YPD solid medium amended with 100 mg/L chloramphenicol. Plates were incubated aerobically at 30 °C for 48 h for subsequent enumeration of colony-forming units (CFU).

### 2.6. Characteristic Spectrum of Dough Fermentation and Acidification

The pH and total titratable acidity (TTA) of liquid sourdough samples were determined according to the method by Plessas et al. [17].

Based on the methodology described by Debonne et al., sourdough aliquots (5 g) were homogenized with 20 mL of 0.1% (*v*/*v*) phosphoric acid aqueous solution and magnetically stirred (500 rpm) for 20 min at ambient temperature [18]. The mixture was centrifuged at 10,000× *g* for 10 min (4 °C), after which the precipitate was discarded. The supernatant was vortex-mixed for 30 s, and a 1 mL aliquot was transferred to a microcentrifuge tube. Following the addition of 20 μL of 20% (*v*/*v*) trichloroacetic acid, samples were incubated at 4 °C for 30 min. Subsequent centrifugation at 10,000× *g* for 20 min (4 °C) was performed, and the resulting supernatant was filtered through a 0.22 μm nylon membrane before storage at 4 °C pending analysis.

Chromatographic separation was achieved using a Shimadzu Shim-pack GIST C18 column (4.6 × 250 mm, 5 μm particle size; Shimadzu Corporation, Kyoto, Japan) under isocratic conditions with a mobile phase consisting of 0.1% (*v*/*v*) aqueous phosphoric acid and methanol (97:3, *v*/*v*). The flow rate was maintained at 1.0 mL/min with UV detection at 210 nm. Analyses were performed at a column temperature of 30 °C using a 20 μL injection volume.

### 2.7. LC–MS Analysis

Based on the fermentation stages defined in Section 2.4, six samples of each were prepared according to the protocol detailed in Table 2. The resulting samples were subsequently contracted to Shanghai Meiji Biomedical Technology Co., Ltd. (Shanghai, China) for liquid chromatography-mass spectrometry (LC-MS) analysis.

Based on metabolomic profiling, sample comparisons were performed through multivariate statistical analysis using Principal Component Analysis (PCA) and Orthogonal Partial Least Squares Discriminant Analysis (OPLS-DA). Metabolic features were annotated against the Genomes (KEGG) database (Release 106.0, https://www.kegg.jp/kegg/pathway.html accessed on 31 December 2025) to identify pathway-associated differential metabolites. Pathway enrichment analysis was conducted via Fisher’s exact test implemented in SciPy.stats, with biological pathway significance determined at *p* < 0.05.

### 2.8. Preparation of Mantou

Based on the standardized protocol detailed in Table 3, after thorough ingredient mixing, kneading was performed in a spiral dough mixer for 10 min (HMJ-A35A1, Bear Kitchen Appliances Co., Ltd., Guangdong, Foshan, China). The dough was subsequently pressed seven times, divided, and molded into cylindrical shapes, and proofed at 30 °C and 80% RH for a specified duration. Steam cooking was executed in a commercial steamer at 100 °C for 25 min, followed by a 5 min standing time before removal. Final products were cooled to ambient temperature (25 ± 1 °C) for 60 min, yielding standardized Mantou.

### 2.9. Comparison of In Vitro Starch Digestion Characteristics of Mantou

#### 2.9.1. Gastrointestinal In Vitro Digestive Simulation

Based on established methodologies [19,20], in vitro gastrointestinal digestion was performed with the following modifications: Mantou powder (1 g) was homogenized with 3 mL of simulated salivary fluid (SSF) containing 75 U/mL α-amylase and incubated at 37 °C for 2 min with constant agitation. Following oral phase digestion, 4 mL of simulated gastric fluid (SGF) was added, pH adjusted to 1.5, supplemented with 2000 U/mL pepsin, and incubated at 37 °C for 2 h with orbital shaking. Subsequently, 8 mL of simulated intestinal fluid (SIF) was introduced, pH adjusted to 7.2, amended with 200 U/mL pancreatin and 10 mM bile salts, and digested at 37 °C for 2 h. Aliquots were collected at 30 min intervals during intestinal digestion, immediately quenched in boiling water for 10 min, and centrifuged at 4500× *g* for 10 min at 4 °C. Reducing sugar content in supernatants was quantified using 3,5-dinitrosalicylic acid (DNS) reagent at 540 nm.

#### 2.9.2. Determination of Total Starch Content (TS) and Free Glucose (FG) Content in Mantou

The total starch (TS) and free glucose (FG) content in Mantou were determined using the method described by Sandhu and Singh [21]. A conversion factor of 0.9 was used to convert glucose into starch equivalents. This method assumes complete hydrolysis of starch to glucose. The calculation formulas are as follows:

(3) $$\mathrm{FG}=\frac{\mathrm{Glucose}\;\mathrm{content}\;\mathrm{in}\;\mathrm{supernatant}\;(\mathrm{mg})}{Sample\;weight\;(g)}$$

(4) $$\mathrm{TS}=\frac{\left(\mathrm{Glucose}\;\mathrm{content}\;\mathrm{in}\;\mathrm{supernatant}\times 0.9-\mathrm{FG}\times \mathrm{Sample}\;\mathrm{weight}\right)}{Sample\;weight}$$

#### 2.9.3. Determination of Digested Starch Types

Referring to the method described by Tang et al. [22], the content of different starch fractions in Mantou was calculated according to the following formulas:

(5) $$\mathrm{RDS}=\frac{(G_{20\min}-\mathrm{FG})\times 0.9}{\mathrm{Quality}\;\mathrm{of}\;\mathrm{Mantou}}\times 100$$

(6) $$\mathrm{SDS}=\frac{(G_{120\min}-G_{20\min})\times 0.9}{\mathrm{Quality}\;\mathrm{of}\;\mathrm{Mantou}}\times 100$$

(7) $$RS=\frac{TS-RDS-SDS}{Quality\;of\;Mantou}\times 100$$

The starch digestion fractions used in this study include rapidly digestible starch (RDS), slowly digestible starch (SDS), and resistant starch (RS).

### 2.10. Determination of Antioxidant Characteristics of Mantou

#### 2.10.1. Preparation of Methanol Extract

Mantou (4 g) was mixed with 40 mL of an 80% (*v*/*v*) methanolic-water solution and homogenized. The mixture was then subjected to ultrasonication for 30 min, followed by centrifugation at 4000× *g* for 15 min. The resulting supernatant was collected as the extract.

#### 2.10.2. Determination of Total Phenolic Content (TPC)

The Folin–Ciocalteu assay was performed as previously described [23]. The sample solution was diluted 10-fold with distilled water. For TPC determination, 1 mL of the diluted sample was mixed with 0.5 mL of 0.25 mol/L Folin–Ciocalteu reagent and vortexed thoroughly. After 5 min, 5 mL of 7% (*w*/*v*) sodium carbonate solution was added, and the mixture was incubated in the dark for 90 min. Absorbance was measured at 760 nm using a spectrophotometer.

#### 2.10.3. Determination of the Radical Scavenging Ability of DPPH and ABTS

For DPPH radical scavenging activity analysis, 1 mL of the extract was mixed with 1 mL of 0.1 mmol/L DPPH solution (prepared in anhydrous methanol). The mixture was vortexed thoroughly and incubated in the dark for 30 min before measuring the absorbance at 517 nm, following a previously reported method [24].

(8) $$\mathrm{DPPH}\;\mathrm{radical}\;\mathrm{scavenging}\;\mathrm{rate}\;(\%)=(1-\frac{A_{2}-A_{1}}{A_{0}})\times 100$$

where A_2_, A_1_, and A_0_ represent the absorbance values of different reaction mixtures: A_2_ is 1 mL of DPPH solution + 1 mL of sample solution, A_0_ is 1 mL of DPPH solution + 1 mL of methanol (80%), A_1_ is 1 mL of methanol (80%) + 1 mL of sample solution.

The ABTS radical scavenging activity was measured using a commercial assay kit in accordance with the manufacturer’s protocol.

#### 2.10.4. Determination of the Reducing Power

1.0 mL of the extract was combined with 1.0 mL of PBS buffer (0.2 mol/L, pH 6.6) and 1.0 mL of 1% (*w*/*v*) potassium ferricyanide solution. The mixture was vortexed and incubated in a water bath at 50 °C for 20 min. The reaction was terminated by adding 1.25 mL of 10% (*w*/*v*) trichloroacetic acid. Subsequently, 1.0 mL of the reaction solution was mixed with 0.5 mL of 0.1% (*w*/*v*) FeCl_3_ and 1.0 mL of distilled water. After 10 min of incubation at room temperature, absorbance was measured at 700 nm, following a previously reported method [25]. The standard curve was prepared using vitamin C (VC) as the reference standard. The total reducing capacity is expressed as the VC equivalent per 100 g of dry matter (mg VC/100 g DM).

### 2.11. Statistical Analysis

All statistical analyses and data visualization were conducted using GraphPad Prism 8.0 and IBM SPSS 27. Statistical significance was defined at *p* < 0.05.

## 3. Results and Discussion

### 3.1. The Analysis of Gas Production Characteristics and Biomass Accumulation

**Figure 1:**
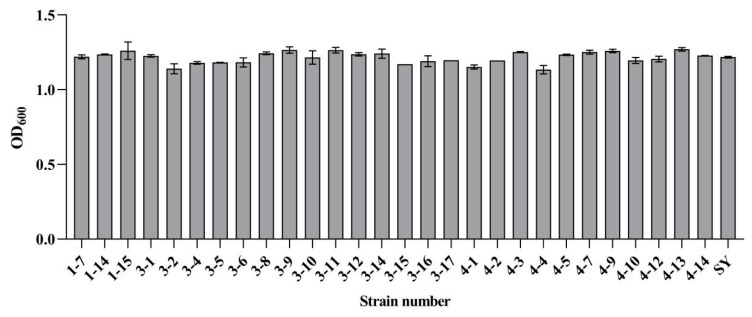
Biomass accumulation of different strains after 24 h of cultivation.

Gas production capacity, a critical determinant of sourdough fermentation kinetics, significantly influences the quality attributes of Mantou. As shown in Table 4, preliminary screening identified 28 strains of *K. humilis* capable of fermentative gas production. While all strains produced sufficient gas to fill Durham tubes within 72 h, commercial *S. cerevisiae* (SY) achieved comparable gas accumulation within 12 h, indicating superior fermentation efficiency. Notably, seven *K. humilis* strains exhibited intermediate gas production rates, filling Durham tubes within 12–24 h. Biomass accumulation, assessed by OD600 measurements after 24 h of growth, revealed variations among strains, ranging from 1.134 (*K. humilis* 4-4) to 1.281 (*K. humilis* 1-5) (Figure 1). Previous studies have demonstrated that different *K. humilis* strains exhibit distinct growth behaviors and interactions in sourdough ecosystems. For instance, variations in growth kinetics and microbial associations have been observed among different *K. humilis* strains when co-cultured with *Fructilactobacillus sanfranciscensis*, indicating strain-specific adaptation and interaction patterns [26]. Based on combined evaluation of gas production kinetics and biomass yield, six strains (*K. humilis* 1-14, 1-15, 3-8, 3-11, 4-3, and 4-14) were selected for further investigation.

### 3.2. The Analysis of Physiological Functional Characteristics of Strains

Sourdough fermentation involves a complex microbial ecosystem, where LAB, acetic acid bacteria, and yeasts metabolically produce organic acids (such as lactic acid and acetic acid) through their metabolic activities, lowering dough pH and modulating yeast fermentation kinetics. Consequently, acid tolerance is a critical trait for yeasts in sourdough applications [27].

**Figure 2:**
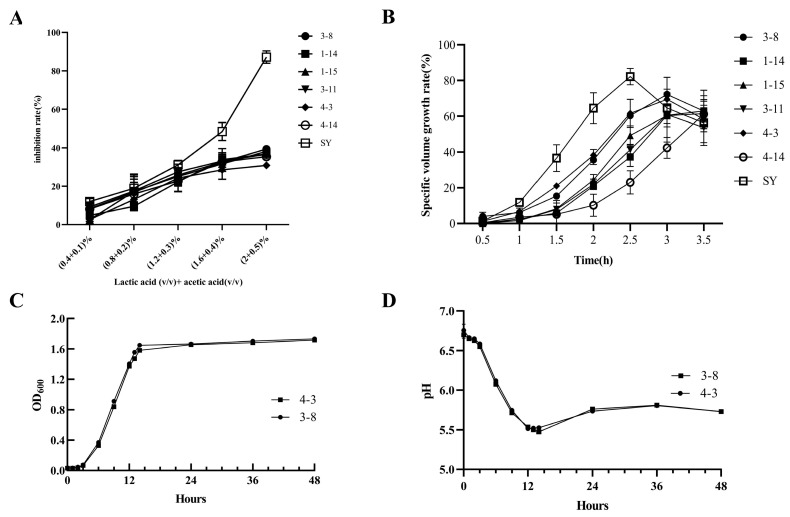
Lactic acid and acetic acid tolerance of different strains (**A**), Dough volume growth rate of different strains (**B**), Growth curve (**C**), pH change (**D**).

As shown in Figure 2A, all seven *K. humilis* strains exhibited inhibition rates similar to *S*. *cerevisiae* at lactic/acetic acid concentrations <0.005 (*v*/*v*). Inhibition rates increased uniformly across strains with rising acid concentrations. Notably, at concentrations >0.02 (*v*/*v*), *K. humilis* strains displayed significantly lower inhibition rates than SY (*p* < 0.05), demonstrating superior acid tolerance and suggesting enhanced compatibility with LAB co-cultures. This finding is consistent with previous reports showing that, compared with *S*. *cerevisiae*, *K. humilis* displays enhanced coexistence with *Lactiplantibacillus plantarum*, which may be attributed to its higher acid tolerance and distinct nutrient competition strategies [28].

Carbon dioxide produced during yeast fermentation is the primary driving force for dough expansion. Using the commercial yeast SY as a control, the leavening capacity of seven yeast strains was evaluated, with the dough volume increase rate used as an indicator of fermentation performance. As shown in Figure 2B, during the initial fermentation stage (0–1.5 h), *K. humilis* exhibited a relatively low fermentation rate, likely due to its adaptation to the new environment and limited metabolic activity during the lag phase. After 1.5 h, the dough volume increased rapidly, indicating active cell proliferation and vigorous growth. Compared with the control, *K. humilis* showed a relatively slower fermentation rate, reaching a maximum volume increase of 72.19% at 3 h, whereas SY achieved a higher maximum of 82.20% at 2.5 h. Notably, different *K. humilis* strains displayed distinct fermentation capacities. During the mid-fermentation stage (1.5–3 h), *K. humilis* 3-8 and 4-3 produced greater dough expansion than the other strains. CO_2_ production in sourdough is mainly driven by yeast metabolism and varies among species, typically following the order *S. cerevisiae* > *K. humilis* > *W. anomalus* [29].

As shown in Figure 2C, *K. humilis* exhibited a lag phase (0–4 h), followed by a logarithmic phase (4–13 h), and stabilized after 13 h. No decline phase occurred within 48 h. Figure 2D demonstrates that during fermentation, the culture medium pH gradually decreased with yeast growth, reaching its lowest point at 13 h. Subsequently, the pH increased slightly from 13 to 36 h before decreasing again after 36 h. *K. humilis* 3-8 and 4-3 showed no significant differences in morphological structure or growth characteristics; Thus, *K. humilis* 3-8 was selected for subsequent experiments.

### 3.3. Changes in Yeast and LAB Counts During Fermentation

To evaluate microbial interactions during dough fermentation, *K. humilis* 3-8 and *F. sanfranciscensis* 5 were co-inoculated at ratios of 1:100 and 1:1 (CFU/g). Two fermentation protocols were tested: (1) simultaneous inoculation of both strains, and (2) delayed inoculation of *F. sanfranciscensis* 5 after 3 h of *K. humilis* 3-8 pre-fermentation.

**Figure 3:**
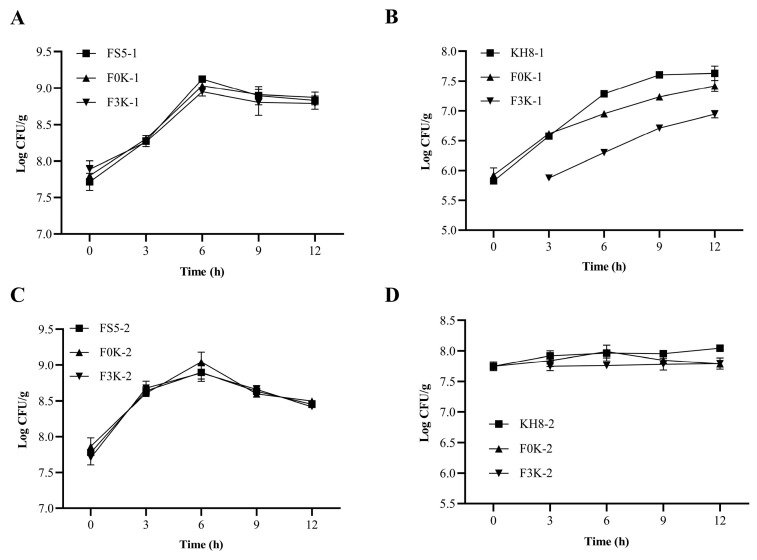
Trend of *F. sanfranciscensis* 5 changes at inoculation ratios of 1:100 (**A**), Trend of *K. humilis* 3-8 changes at inoculation ratios of 1:100 (**B**), Trend of *F. sanfranciscensis* 5 changes at inoculation ratios of 1:1 (**C**), Trend of *K. humilis* 3-8 changes at inoculation ratios of 1:1 (**D**).

As shown in Figure 3, the population dynamics of *F. sanfranciscensis* 5 in co-cultures mirrored those in monocultures, regardless of inoculation ratio or sequence. During 0–6 h, *F. sanfranciscensis* 5 exhibited rapid growth (Figure 3A,C), peaking at 8.4 log CFU/g (1:1) and 8.8 log CFU/g (1:100) by 12 h—a 100-fold increase from initial inoculation (*p* > 0.05 vs. controls). These results confirm that *K. humilis* 3-8 does not suppress *F. sanfranciscensis* 5, which demonstrates robust dough adaptability. *K. humilis* does not confer a population advantage in co-culture with LAB and does not significantly influence their growth, supporting the view that microbial coexistence in sourdough is largely governed by competitive interactions rather than strict mutualism [30].

In contrast, *K. humilis* 3-8 growth varied with fermentation conditions (Figure 3B,D). At a 1:1 ratio, its population in control dough (KH8-2) increased steadily, while in F0K-2 dough (simultaneous inoculation), growth increased after 6 h, likely due to organic acid accumulation from *F. sanfranciscensis* 5. In F3K-2 dough (delayed inoculation), cell counts stabilized, possibly inhibited by pre-existing acidity. At a 1:100 ratio, *K. humilis* 3-8 growth in F0K dough diverged significantly from controls after 3 h (*p* < 0.05), indicating progressive inhibition by *F. sanfranciscensis* 5. Based on these findings, a 1:100 inoculation ratio (10^5^:10^7^ CFU/g of *K. humilis* 3-8: *F. sanfranciscensis* 5) was selected for optimal microbial balance.

### 3.4. The Analysis of the Characteristic Spectrum of Dough Fermentation and Acidification

**Figure 4:**
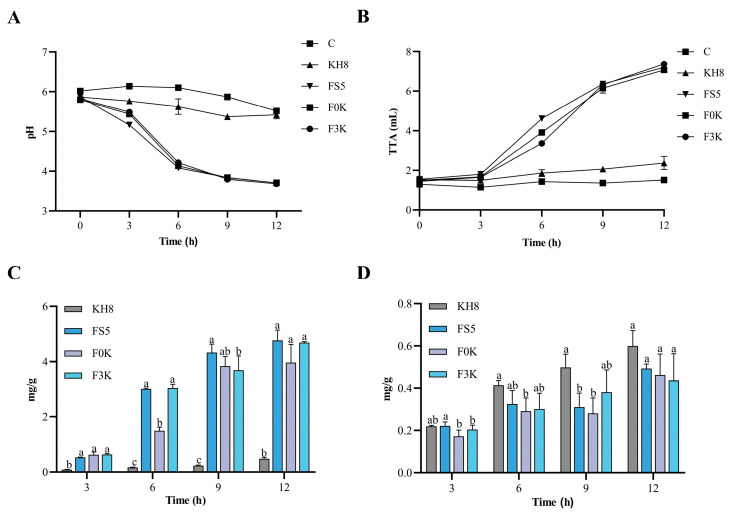
Trend of pH changes in sourdough (**A**), trend of TTA changes in sourdough (**B**), trend of lactic acid content changes in sourdough (**C**), trend of acetic acid content in sourdough (**D**). Different letters indicate a significant difference between different groups at the same time point (*p* < 0.05).

The pH and TTA of dough are critical parameters influencing fermentation dynamics. Acidic conditions enhance gluten network formation and modify dough rheology, ultimately determining the textural and sensory properties of baked products [31,32]. As shown in Figure 4A,B, pH and TTA profiles during co-fermentation of *K. humilis* 3-8 and *F. sanfranciscensis* 5 closely mirrored those of *F. sanfranciscensis* 5 monocultures. The rapid pH decline and TTA increase observed in mixed fermentations were driven primarily by lactic and acetic acid production from *F. sanfranciscensis* 5 metabolism, whereas *K. humilis* 3-8 monocultures showed minimal pH/TTA changes (*p* > 0.05). Notably, *F. sanfranciscensis* 5 monocultures exhibited faster acidification rates than co-cultures, likely due to alcohol-organic acid esterification by *K. humilis* 3-8, reducing free acid concentrations [9].

Organic acids modulate sourdough shelf-life and sensory attributes. As demonstrated by Verdonck et al. [33], lactic acid accelerates fermentation, while acetic acid inhibits microbial activity below pH 4.9. Acetic acid (pKa = 4.76) exhibits pH-dependent antimicrobial activity. Under the typical sourdough pH conditions (3.5–4.5), a considerable fraction of acetic acid remains undissociated, thereby enhancing its antimicrobial effectiveness [18]. In this study, lactic acid accumulation (Figure 4C) was significantly lower in *K. humilis* 3-8 monocultures (KH8) than in other doughs (*p* < 0.05), confirming *F. sanfranciscensis* 5 as the dominant acid producer. After 6 h, lactic acid levels in F0K co-cultures were 23% lower than in *F. sanfranciscensis* 5 monocultures (FS5) (*p* < 0.05), suggesting alcohol-mediated acid conversion into flavor esters. Acetic acid concentrations remained stable across all conditions (Figure 4D), indicating lactic acid’s predominant role in sourdough acidification—consistent with its lower pKa (3.86 vs. 4.76 for acetic acid) and higher proton-donating capacity [33].

### 3.5. Metabolomic Analysis

**Figure 5:**
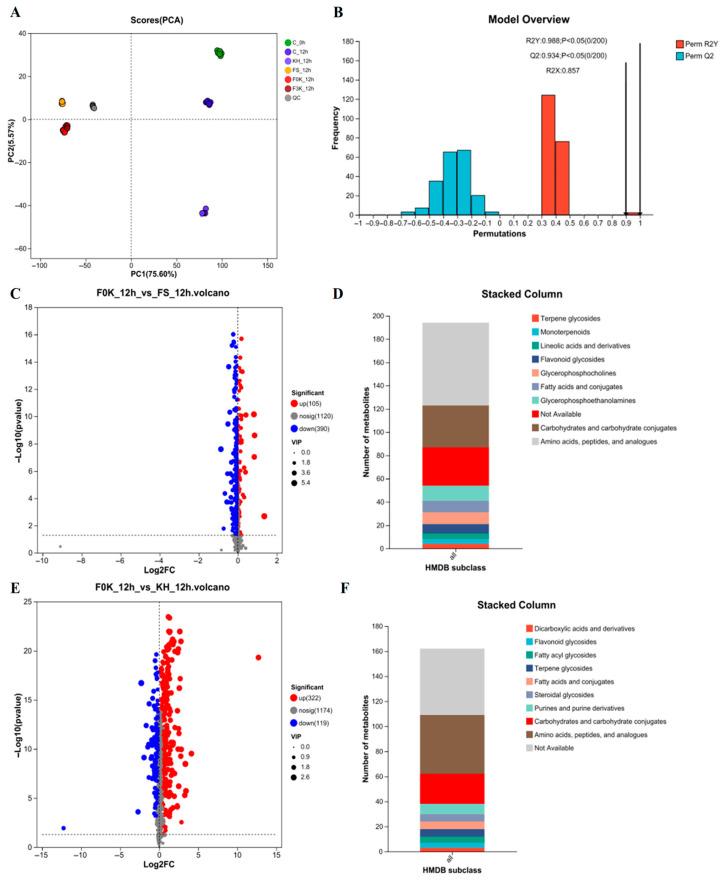
PCA score charts (**A**), PLS-DA permutation test plots (**B**), F0K_12h_vs_FS_12h volcanic map of differential metabolites (**C**), Statistical chart of classification and distribution of F0K_12h_vs_FS_12h HMDB compounds (**D**), F0K_12h_vs_KH_12h volcanic map of differential metabolites (**E**), Statistical chart of classification and distribution of F0K_12h_vs_KH_12h HMDB compounds (**F**).

Untargeted metabolomics analysis identified 977 and 638 metabolites in positive and negative ion modes, respectively. Principal component analysis (PCA), an unsupervised multivariate statistical method, was employed to evaluate global metabolic variations among sample groups and assess data quality. Quality control (QC) samples clustered tightly within the 95% confidence interval (Figure 5A), demonstrating system stability. The first two principal components (PC1 and PC2) explained 75.60% and 5.57% of total variance, respectively, with a cumulative contribution of 81.17%, confirming robust metabolic discrimination between groups.

Partial least squares-discriminant analysis (PLS-DA) was subsequently performed to model relationships between metabolite profiles and fermentation conditions. As shown in Figure 5B, the model exhibited high validity, with interpretation rates (R^2^X = 0.857 for metabolites; R^2^Y = 0.988 for group classification) and predictive ability (Q^2^ = 0.934) approaching unity, indicating neither overfitting nor bias. These results confirm the model’s suitability for identifying differentially abundant metabolites.

#### 3.5.1. The Analysis of Differential Metabolites and Metabolic Pathways Between Single-Strain and Mixed-Strain Fermentation

To elucidate the metabolic interactions between *K. humilis* 3-8 and *F. sanfranciscensis* 5 in liquid sourdough, we conducted comparative metabolomic analysis of KH_12h, FS_12h, and F0K_12h fermentation groups. Using OPLS-DA modeling with VIP > 1 and *p* < 0.05 as selection criteria, we identified 495 significantly differential metabolites in F0K_12h versus FS_12h comparisons (Figure 5C), comprising 105 upregulated and 390 downregulated compounds. HMDB database annotation classified 310 metabolites (Figure 5D), with amino acids/peptides/analogues (22.9%) and carbohydrates/complexes (11.61%) representing the predominant classes. Similarly, F0K_12h versus KH_12h comparisons revealed 441 differential metabolites (322 up-regulated, 119 down-regulated; Figure 5E), again dominated by amino acid derivatives (18.08%) and carbohydrates (9.23%; Figure 5F).

KEGG pathway analysis demonstrated significant enrichment (*p* < 0.05) in amino acid metabolic pathways, including phenylalanine metabolism and alanine/aspartate/glutamate metabolism. Notably, key amino acids (L-aspartate, L-methionine, L-tyrosine, etc.) were downregulated in co-cultures, suggesting dynamic nitrogen-source utilization: *K. humilis* preferentially consumed free amino acids, while *F. sanfranciscensis* acid-activated flour proteases and secreted proteases to replenish the amino acid pool via gluten degradation. This metabolic cross-feeding aligns with established models of yeast-LAB interactions [34], where mixed fermentation balances yeast consumption against bacterial proteolysis. The observed amino acid flux directly impacts flavor development through conversion to volatile aldehydes, ketones, and sulfur compounds [35]. The release of free amino acids, such as phenylalanine, leucine, cysteine, and ornithine, contributes not only to microbial nitrogen supply but also to flavor development in bread. These amino acids act as key precursors of volatile compounds and can be metabolized via pathways such as the Ehrlich pathway to generate aldehydes, alcohols, ketones, and sulfur-containing compounds.

Furthermore, carbohydrate metabolism pathways showed coordinated upregulation in F0K_12h versus FS_12h (*p* < 0.05), including central carbon metabolism (pyruvate metabolism, glycolysis/gluconeogenesis, TCA cycle), pentose phosphate pathway, branch-chain acid metabolism (C5-dibasic acids, butanoate), and sugar interconversions (pentose/glucuronate, ascorbate/aldehyde, glyoxylate/dicarboxylate), collectively supporting enhanced biomass production and biosynthetic capacity in the co-culture system.

#### 3.5.2. The Analysis of the Impact of Delayed Yeast Inoculation on Dough

**Figure 6:**
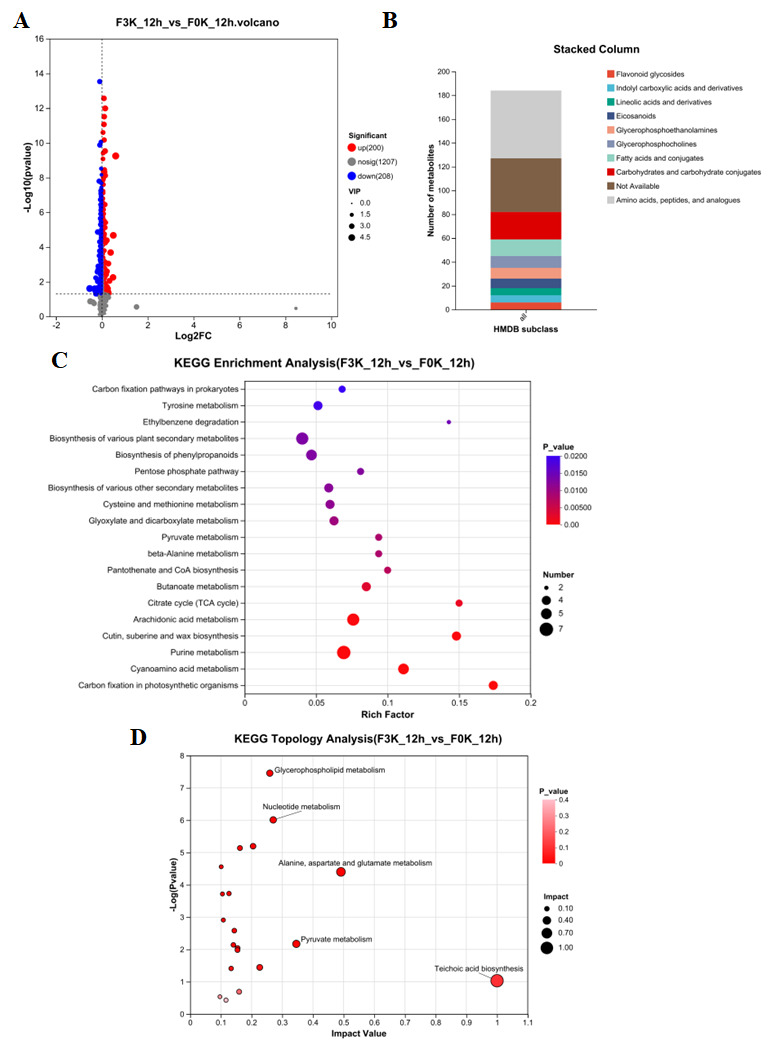
F3K_12h_vs_F0K_12h volcanic map of differential metabolites (**A**), Statistical chart of classification and distribution of F3K_12h_vs_F0K_12h HMDB compounds (**B**), KEGG enrichment analysis chart (**C**), KEGG topology analysis bubble chart (**D**).

Comparative metabolomic analysis between F3K_12h (sequential inoculation) and F0K_12h (simultaneous inoculation) liquid sourdough systems identified 408 significantly differential metabolites (200 upregulated and 208 downregulated) through non-targeted metabolic profiling (Figure 6A). HMDB database annotation of these metabolites revealed that 260 compounds could be classified, with amino acids/peptides/analogues (18.94%) and carbohydrates/complexes (7.64%) representing the predominant metabolite classes in F3K_12h (Figure 6B). Pathway enrichment and topological analysis identified seven significantly altered metabolic pathways (*p* < 0.05), including carbon sequestration, cyanoamino acid metabolism, purine metabolism, citrate cycle (TCA cycle), butyrate metabolism, pyruvate metabolism, and cysteine/methionine metabolism in photosynthetic organisms (Figure 6C,D). Notably, the observed downregulation of key carbohydrate metabolism pathways (TCA cycle, butyrate metabolism, and pyruvate metabolism) in F3K_12h suggests that metabolic byproducts generated during *F. sanfranciscensis* 5 growth may inhibit the central carbon metabolism of *K. humilis* 3-8, thereby restricting yeast proliferation in sequentially inoculated fermentation systems. This metabolic suppression provides mechanistic insight into the growth dynamics observed between different inoculation protocols.

#### 3.5.3. Metabolic Difference Analysis of Four Fermentation Methods

**Figure 7:**
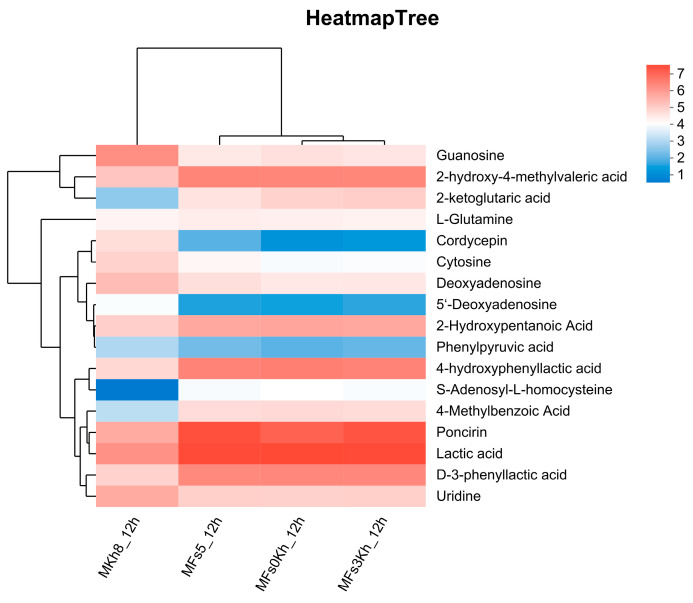
Heatmap and cluster analysis of four different groups of differential metabolite.

Metabolic profiling revealed distinct metabolite distribution patterns across the four fermentation systems, shaped by strain combinations and inoculation strategies (Figure 7). In MFs5_12h, *F. sanfranciscensis* dominated homolactic fermentation under oxygen-limited conditions, with elevated lactate dehydrogenase activity driving efficient glucose-to-lactate conversion [36]. Conversely, MKh8_12h exhibited respiratory metabolism characteristic of *K. humilis*, where TCA cycle activation promoted glutamate dehydrogenase-mediated production of 2-ketoglutarate from glutamate. However, succinate dehydrogenase inhibition due to limited mitochondrial CoA availability led to intermediate accumulation, contributing unique flavor profiles [37]. The simultaneously inoculated MFs0Kh_12h system showed marked S-adenosyl-L-homocysteine accumulation, reflecting microbial competition-induced metabolic stress. Here, yeast-mediated vitamin B12 depletion impaired S-adenosyl-L-homocysteine hydrolase activity in LAB [38], while oxidative stress further blocked its degradation into adenosine and homocysteine. In contrast, the delayed inoculation strategy (MFs3Kh_12h) triggered yeast activation of branched-chain amino acid compensation pathways in response to LAB-induced acidification. Through highly active aminotransferases, valine and phenylalanine were converted to 2-ketoglutarate and phenylpyruvate, respectively, with subsequent yeast-specific reductase-mediated formation of 2-hydroxypentanoate and D-3-phenyllactate [39]. The latter’s derivative, benzoic acid, conferred synergistic antimicrobial activity [40]. These findings demonstrate how strain specificity, inoculation timing, and microbial cross-talk collectively modulate metabolic flux through carbon partitioning, cofactor competition, and stress adaptation mechanisms.

### 3.6. The Analysis of In Vitro Digestion Characteristics of Starch in Different Mantou

**Figure 8:**
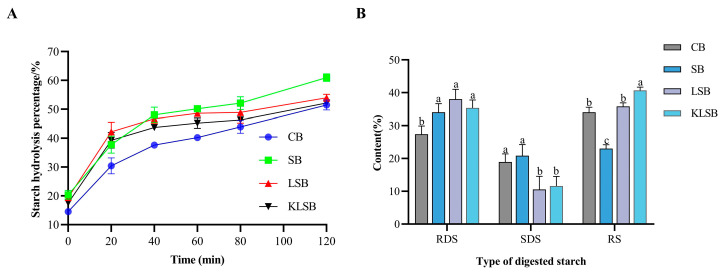
Starch hydrolysis percentage curves of different Mantou (**A**), Content of different types of digested starch in various Mantou (**B**). Different letters above the bars indicate significant differences between group CB, SB, LSB, and KLSB (*p* < 0.05).

Starch digestion characteristics of Mantou were significantly influenced by liquid sourdough fermentation (Figure 8). While all four Mantou types exhibited similar in vitro starch digestion kinetics with progressively increasing hydrolysis rates over time (Figure 8A), marked differences emerged in starch fraction distribution (Figure 8B). The rapid digestible starch (RDS, digested within 20 min) content increased significantly from 27.40% in control Mantou to 34.01–38.01% in fermented samples (*p* < 0.05), demonstrating enhanced starch accessibility through fermentation [41,42]. Notably, the mixed-culture fermented KLSB (35.34% RDS) showed intermediate values between LAB-fermented LSB (38.01%) and control SB (34.01%), suggesting microbial composition modulates digestion kinetics. Slowly digestible starch (SDS, 20–120 min digestion) fractions decreased dramatically from 20.85% in SB to 10.57–11.61% in liquid sourdough-fermented samples (*p* < 0.05), indicating fermentation preferentially alters intermediate digestion-rate starch structures. Most remarkably, resistant starch (RS, undigested after 120 min) content nearly doubled, increasing from 23.02% in SB to 35.80% (LSB) and 40.68% (KLSB) (*p* < 0.05). The elevated RS fraction suggests potential health benefits, including moderated glycemic response and enhanced colonic fermentation [42], while the superior performance of KLSB highlights the synergistic effects of yeast-LAB co-cultures on starch modification.

Starch digestibility in cereal foods is modulated by multiple physicochemical and physiological factors, including structural organization at macro-scales and micro-scales, enzyme-substrate interactions, gastrointestinal motility, endogenous enzyme inhibitors, and luminal viscosity [43,44]. In the present study, fermentation with *F. sanfranciscensis* 5 generated substantial organic acids that collectively influenced starch digestion dynamics: lactic acid directly impaired α-amylase activity, while acetic and propionic acids delayed gastric emptying [45]. These acidic metabolites not only lowered dough pH (promoting uniform crumb structure formation) but also contributed to reduced glycemic response, as demonstrated by the decreased glycemic index in fermented Mantou [46,47]. Furthermore, fermentation-derived phenolic compounds formed starch-polyphenol complexes that sterically hindered enzymatic hydrolysis [48]. The synergistic action of these acid-mediated and polyphenol-dependent mechanisms explains the superior starch modification achieved with mixed-culture liquid sourdough compared to conventional fermentation approaches.

### 3.7. The Analysis of the Antioxidant Properties of Different Mantou

**Figure 9:**
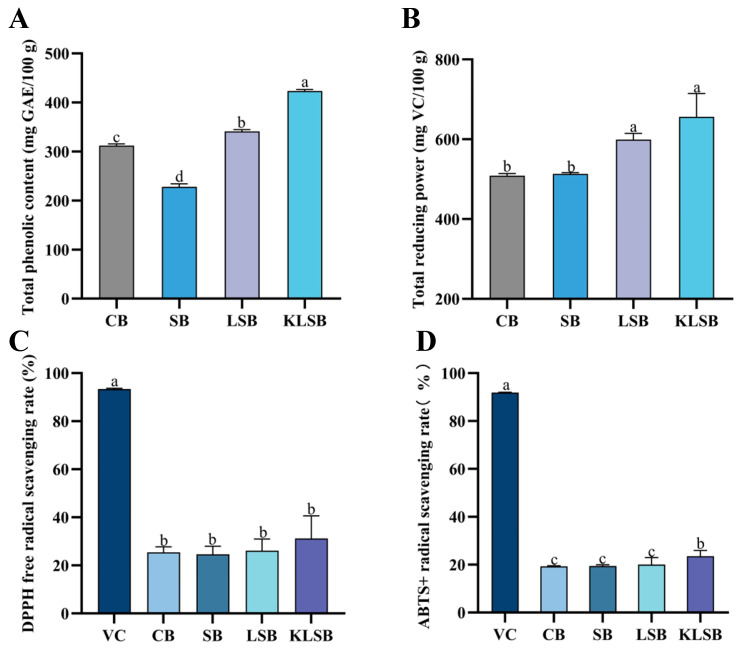
Total phenolic content (**A**), total reducing power (**B**), DPPH radical scavenging rate (**C**), ABTS radical scavenging rate (**D**) of different mantou. GAE, gallic acid equivalent; VC, vitamin C; CB, plain mantou without fermentation starter; SB, mantou with commercial yeast; LSB, mantou with liquid sourdough FS5; KLSB, mantou with liquid sourdough F0K. Different letters above the bars indicate significant differences between group VC, CB, SB, LSB, and KLSB (*p* < 0.05).

Building upon established evidence that the co-culture of *K. humilis* 3-8 and *F. sanfranciscensis* 5 enhances antioxidant capacity in liquid sourdough, we systematically evaluated the antioxidant properties of sourdough-fermented Mantou through four key metrics. As shown in Figure 9, conventional *S. cerevisiae* fermentation significantly reduced total phenolic content (TPC) in SB Mantou compared to the blank control (*p* < 0.05). In contrast, SY fermentation showed no measurable impact on three other antioxidant indicators: total reducing power (0.82 ± 0.04 vs. 0.85 ± 0.03 mmol TE/g, *p* > 0.05), DPPH radical scavenging capacity (48.7% ± 2.1% vs. 51.3% ± 1.8%, *p* > 0.05), and ABTS scavenging activity (63.5% ± 3.2% vs. 65.1% ± 2.9%, *p* > 0.05). These results demonstrate that while SY fermentation depletes certain phenolic compounds, it fails to significantly alter the overall antioxidant profile of Mantou, highlighting the need for alternative fermentation strategies to enhance functional properties.

Comparative analysis of antioxidant properties revealed significant enhancement in liquid sourdough-incorporated Mantou (Figure 9). Both LSB and KLSB exhibited markedly increased total phenolic content (52.63% and 92.11% increase, respectively) and total reducing power (16.82% and 27.89% increase, respectively) compared to conventional SB Mantou (*p* < 0.05; Figure 9A,B). Radical scavenging assays demonstrated parallel improvements, with DPPH and ABTS scavenging capacities in LSB and KLSB significantly surpassing SB values (Figure 9C,D). Notably, KLSB outperformed LSB in both total phenolics and ABTS scavenging capacity (*p* < 0.05), confirming the synergistic antioxidant effect between *K. humilis* 3-8 and LAB previously observed in liquid sourdough systems. However, despite these improvements, all Mantou samples showed significantly lower antioxidant activity than their corresponding liquid sourdough precursors (*p* < 0.05), likely due to thermal degradation of heat-sensitive antioxidants (e.g., polyphenols and B vitamins) during the steaming process [49]. These findings collectively demonstrate that while liquid sourdough fermentation substantially enhances Mantou’s antioxidant profile, processing-induced losses highlight the need for thermal stabilization strategies to maximize retention of bioactive compounds.

## 4. Conclusions

This study demonstrates that the selected strains *K. humilis* 3-8 and *F. sanfranciscensis* 5 exhibit remarkable synergistic potential in liquid sourdough co-fermentation, characterized by dynamic interactions in carbon and nitrogen metabolism. While *F. sanfranciscensis* 5 dominated dough acidification and modulated the carbon metabolism of *K. humilis* 3-8 through metabolic cross-talk, their combined application in Mantou production yielded significant quality improvements, including a 77% increase in resistant starch content and 92% enhancement in antioxidant capacity compared to conventional products. The developed liquid sourdough starter not only surpassed commercial Mantou in physicochemical and nutritional profiles but also elucidated the fundamental microbial interaction mechanisms governing cereal fermentations. These findings not only resolve the long-standing challenge of standardizing traditional sourdough for industrial scale-up but also provide mechanistic insights into yeast-LAB metabolic cross-talk that will inform the next generation of functional cereal fermentations. Future work should extend this approach to diverse grain matrices and validate long-term process stability under industrial conditions.

## Figures and Tables

**Table 1:** Formulations of liquid sourdough in different groups.

Sample Name	Flour (g)	Water (g)	*K. humilis* 3-8 (CFU/g)	*F. sanfranciscensis* 5 (CFU/g)
C	100	125	-	-
KH8	100	125	10^5^	-
FS5	100	125	-	10^7^
F0K	100	125	10^5^	10^7^
F3K	100	125	10^5^	10^7^
KH8-2	100	125	10^7^	-
FS5-2	100	125	-	10^7^
F0K-2	100	125	10^7^	10^7^
F3K-2	100	125	10^7^	10^7^

Note: C, control dough without microbial inoculation; KH8, dough inoculated with *K. humilis* 3-8; FS5, dough inoculated with *F. sanfranciscensis* 5; F0K, dough co-inoculated with *F. sanfranciscensis* 5 and *K. humilis* 3-8 simultaneously at a ratio of 100:1 (LAB:yeast); F3K, dough inoculated with *K. humilis* 3-8 after 3 h fermentation of *F. sanfranciscensis* 5 (LAB:yeast = 100:1); KH8-2, dough inoculated with *K. humilis* 3-8 at a higher level (10^7^ CFU/g); FS5-2, dough inoculated with *F. sanfranciscensis* 5 at 10^7^ CFU/g; F0K-2, dough co-inoculated with *F. sanfranciscensis* 5 and *K. humilis* 3-8 simultaneously at a ratio of 1:1; F3K-2, dough inoculated with *K. humilis* 3-8 after 3 h fermentation of *F. sanfranciscensis* 5 (LAB:yeast = 1:1).

**Table 2:** Sample grouping scheme.

Sample Name	Sampling Method
C_0h	Take samples directly from the unfermented blank dough
C_12h	Take samples after the blank dough has fermented for 12 h
KH_12h	Sampling after 12 h of single strain fermentation with *K. humilis* 3-8
FS_12h	Sampling after 12 h of single strain fermentation with *F. sanfranciscensis* 5
F0K_12h	*K. humilis* 3-8 and *F. sanfranciscensis* 5 were inoculated and fermented for 12 h at the same time before sampling
F3K_12h	The dough was inoculated with *K. humilis* 3-8 and fermented for 3 h. It was then mixed with *F. sanfranciscensis* 5 and fermented for an additional 12 h before sampling

**Table 3:** Formulation of mantou.

Sample	Flour (%)	Water (%)	Commercial *Saccharomyces cerevisiae* (%)	Liquid Sourdough (%)	Proofing Time (min)
CB	100	50	-	-	60
SB	100	50	0.5	-	60
LSB	100	37	0.5	30	80
KLSB	100	37	0.5	30	80

Note: CB, blank mantou; SB, mantou with commercial yeast; LSB, mantou with FS5 liquid sourdough; KLSB, mantou with F0K liquid sourdough.

**Table 4:** Time required for gas production to fill the durham tube by different strains.

Gas Filling Time	Quantity	Strain Number
0–12 h	1	SY
12–24 h	7	*K. humilis* 1-7, 3-5, 3-8, 3-15, 4-1, 4-2, and 4-3
24–36 h	3	*K. humilis* 1-14, 1-15, and 4-14
36–48 h	5	*K. humilis* 3-2, 3-4, 3-6, 3-11, and 4-10
48–60 h	9	*K. humilis* 3-9, 3-10, 3-12, 3-16, 3-17, 4-4, 4-5, 4-9, and 4-12
60–72 h	4	*K. humilis* 3-1, 3-14, 4-7, and 4-13

## Data Availability

The original contributions presented in this study are included in the article. Further inquiries can be directed to the corresponding author.
